# A Novel Monoclonal Antibody to Human Laminin α5 Chain Strongly Inhibits Integrin-Mediated Cell Adhesion and Migration on Laminins 511 and 521

**DOI:** 10.1371/journal.pone.0053648

**Published:** 2013-01-07

**Authors:** Zenebech Wondimu, Shahin Omrani, Taichi Ishikawa, Fawad Javed, Yuko Oikawa, Ismo Virtanen, Erkki Juronen, Sulev Ingerpuu, Manuel Patarroyo

**Affiliations:** 1 Department of Dental Medicine, Karolinska Institutet, Stockholm, Sweden; 2 Institute of Biomedicine/Anatomy, University of Helsinki, Helsinki, Finland; 3 Institute of Biomedicine, University of Tartu, Tartu, Estonia; 4 Institute of Molecular and Cell Biology, University of Tartu, Tartu, Estonia; Biomedical Research Foundation of the Academy of Athens, Greece

## Abstract

Laminins, a large family of αβγ heterotrimeric proteins mainly found in basement membranes, are strong promoters of adhesion and migration of multiple cell types, such as tumor and immune cells, via several integrin receptors. Among laminin α (LMα) chains, α5 displays the widest tissue distribution in adult life and is synthesized by most cell types. Here, we have generated and characterized five novel monoclonal antibodies (mAbs) to the human LMα5 chain to further study the biological relevance of α5 laminins, such as laminins 511 (α5β1γ1) and 521 (α5β2γ1). As detected by ELISA, immunohistochemistry, immunoprecipitation and Western blotting, each antibody displayed unique properties when compared to mAb 4C7, the prototype LMα5 antibody. Of greatest interest, mAb 8G9, but not any other antibody, strongly inhibited α3β1/α6β1 integrin-mediated adhesion and migration of glioma, melanoma, and carcinoma cells on laminin-511 and, together with mAb 4C7, on laminin-521. Accordingly, mAb 8G9 abolished the interaction of soluble α3β1 integrin with immobilized laminins 511 and 521. Binding of mAb 8G9 to laminin-511 was unaffected by the other mAbs to the LMα5 chain but largely hindered by mAb 4E10 to a LMβ1 chain epitope near the globular domain of laminin-511. Thus, mAb 8G9 defines a novel epitope localized at or near the integrin-binding globular domain of the LMα5 chain, which is essential for cell adhesion and migration, and identifies a potential therapeutic target in malignant and inflammatory diseases.

## Introduction

Laminins are a family of large adhesive heterotrimeric molecules composed by disulfide-bonded α, β, and γ chains [1], [2], [3], [4]. These proteins are major components of basement membranes (BMs) and effectors of tissue architecture, but can also be found in other anatomical locations [1], [2], [3], [4]. To date, five α, three β, and three γ laminin chains have been reported in the literature, which assemble into more than 15 laminin isoforms [1], [2], [3], [4]. In the recent nomenclature, laminins are named according to their chain composition [2]. Thus, laminin α4β2γ1, previously called laminin-9, is now denominated laminin-421.

Laminins are synthesized by numerous cell types of solid tissues, and expression of the various laminin isoforms, particularly their α chain, is cell and tissue specific [3], [4], [5]. The prototype laminin-111, originally isolated from a mouse tumor in 1979 [6], has been well characterized biochemically, and much of the *in vitro* functional data ascribed to laminins are based on studies performed with this laminin isoform [1], [3], [4]. However, expression of the laminin α1 (LMα1) chain in adult tissues is highly restricted to a limited subpopulation of epithelial cells [7], [8]. In contrast, the other and more recently described laminin α chains (LMα2-α5), which constitute most laminin isoforms, have a much wider tissue distribution but their actions on cells are less well understood or unknown [3], [4]. The physiological relevance of laminin α chains is illustrated by congenital muscular dystrophy and junctional epidermolysis bullosa, two genetic human diseases of muscle and skin caused by mutations in LMα2 and α3 chains, respectively [1], [3], [4]. Laminins are recognized, through their α chain, by nearly ten different integrins in an isoform –specific fashion and strongly promote cell adhesion and migration [3], [4], [5]. α3β1, α6β1, α7β1 and α6β4 are known as “classical” laminin-binding integrins [9].

LMα5 is the largest of all laminin chains (nearly 3700 amino acids, 350 kDa) and the most widely expressed laminin α chain in adult life [1], [10], [11], [12]. Nevertheless, it was the last laminin α chain to be identified. It constitutes laminin-511 (laminin 10), laminin-521 (laminin 11) and laminin-523 (laminin 15), and is present in epithelial BM (laminin-511) and vascular endothelial BM (laminin-511 and laminin-521), as well as in lymph nodes, lung, kidney and many other organs [1], [2], [3], [4], [13]. Most normal and malignant cells can synthesize and secrete α5-laminins [1], [3], [14]–[24]. Deletion of the LMα5 gene is lethal as deficient mice die during fetal life because of multiple malformations [25]. Among human laminin α chains, LMα5 is unique in containing functional RGD sequences [12], [26]. An RGD sequence found in the short arm of the mouse LMα1 chain is not conserved in human LMα1 [26]. Recent availability of both natural and recombinant laminin-511 has demonstrated that α5-laminins strongly mediate adhesion and migration of a large variety of cell types, including tumor and immune cells, and binding assays have shown direct interaction of isolated α3β1, α6β1, α7β1, α6β4 and αVβ3 integrins with purified laminin-511 [12], [14], [16], [18], [20], [21], [22], [23], [24], [27], [28], [29], [30], [31], [32]. Although α3β1 and α6β1, and probably α7β1 and α6β4, integrins recognize the laminin globular (LG) modules of the LMα5 chain [33], αVβ3 has been reported to bind the RGD-containing IVa module of the short arm of this chain [26]. Lutheran blood group/basal cell adhesion molecule (Lu/BCAM) (CD239), a member of the immunoglobulin superfamily expressed by erythrocytes and some epithelial cells, is another cell surface receptor for laminin-511 and also binds the LG domain of the LMα5 chain [33], [34], [35], [36].

Antibodies are most valuable reagents for analyzing laminins and other proteins. Since the end of the 1970s rabbit polyclonal antibodies to mouse laminin-111 have been extensively used, but these antisera cross-react with most laminin isoforms [1], [3], [4]. In contrast, monoclonal antibodies (mAbs) are mostly laminin-chain specific and can discriminate among laminin isoforms. In spite of this, only a few mAbs against laminin α chains are currently available. In previous studies we generated and characterized several mAbs to the human LMα4 chain [30], [37]. These reagents have been most useful in the analysis of α4 laminins [22], [23], [24], [30], [37].

Immunological reagents to the LMα5 chain are even scantier, in spite of the obvious relevance of α5 laminins in cell and cancer biology. mAb 4C7 is today the prototype antibody to LMα5 [38]. However, this antibody, which was generated by immunization with “laminin” isolated from human placenta, was considered for over a decade to be LMα1 specific, generating confusion in the scientific literature [38], [39]. The mAb 4C7 binds to LG1 of the LMα5 chain and appears to interfere with some functions of α5 laminins, including α6β1 integrin binding, but its ability to inhibit cell adhesion and migration seems to be limited [34], [38], [40], [41] (present study). This antibody is excellent for immunohistochemistry of frozen sections, but its reactivity is reduced in formalin-fixed and paraffin-embedded sections. Furthermore, it works efficiently for immunoprecipitation, but not at all for Western blotting [38], [39] (present study).

In the present study, we report generation and characterization of five novel mAbs to the LMα5 chain. As measured by ELISA, immunohistochemistry, Western blotting and immunoprecipitation, each antibody displays unique properties when compared to mAb 4C7. The mAb 4B5 efficiently reacted in Western blotting and, of greater interest, mAb 8G9 strongly inhibited adhesion and migration of cancer cells on α5 laminins mediated by α3β1 and α6β1 integrins.

## Materials and Methods

### Production of novel mAbs to human LMα5 chain

Female Balb/c mice were first immunized with natural human laminin-511 purified from A549 lung adenocarcinoma cells, as described [14], using Freund's complete adjuvant (Sigma-Aldrich; St. Louis, MO, USA). Three further immunizations were made using Freund's incomplete adjuvant (Sigma-Aldrich) with the same laminin. The last immunization was performed three days before hybridization. Hybridomas were generated according to standard methods [42]. In preliminary screening, hybridomas were tested for reactivity with laminin-511 from A549 cells and placenta laminin-211 (merosin) (Millipore, Billerica, MA, USA) by ELISA. All selected antibodies were mouse immunoglobulin (Ig) G1. In the final screening and in all other assays, recombinant human (rh) laminins 411, 511 and 521 purchased from BioLamina (Stockholm, Sweden) were used. All animal work was conducted according to relevant national and international guidelines, with approval of the Institutional Animal Care and Use Committee of Tartu University and the Estonian Department of Agriculture. Mice were housed in the animal facility, provided standard rodent chow and water *ad libitum*, and sacrificed with carbon dioxide.

### Cell lines

In the present study, the following human cancer cell lines were used: A549 lung adenocarcinoma [14], [43], KG1C glioma [22], BE melanoma [24] and MDA-MB-231 breast carcinoma [44]. Cells were grown and maintained in RPMI-1640 medium with 10% fetal bovine serum and supplemented with HEPES buffer, penicillin and L-glutamine.

### Enzyme-linked immunosorbent assay (ELISA)

For conventional ELISA, 96-well plates (Maxisorp, Nunc, Roskilde, Denmark) were coated for 1 hour at room temperature (RT) with rh laminins 411, 511 and 521 at 2 µg/ml. Following blocking with 0.5% bovine serum albumin (BSA), mAbs were added at a final concentration of 1 μg/ml and allowed to interact for 1 hour at 4°C. After three washes with phosphate buffered saline (PBS), bound antibodies were detected using goat antibodies to mouse Ig coupled to horseradish peroxidase (HRP) (Dako, Glostrup, Denmark) and the enzyme activity was measured using orthophenylenediamine (Sigma-Aldrich). The plates were analyzed using a generic ELISA reader (at 492 nm). In addition to the novel antibodies, mAbs 4C7 (LMα5) [38], 3H2 (LMα4) [30], DG10 (LMβ1) [45] and 22 (LMγ1) (BD Biosciences, Erembodegem, Belgium) [45] were used.

### Immunoprecipitation and sodium dodecyl sulphate (SDS)-polyacrylamide gel electrophoresis (PAGE)/Western blotting

For immunoprecipitation, the primary antibody-antigen complex was produced by mixing 20 µl of mAbs against LMα5 chain (400 µg/ml) with 1 ml of conditioned medium from A549 cells. In parallel, rabbit anti-mouse IgG was added to Protein G Sepharose beads (GE HealthCare Biosciences, Uppsala, Sweden). Both complexes were incubated separately under continuous inversion for 1,5 hour at 4°C. Subsequently, the two complexes were mixed and further incubated for 1,5–2 hours at 4°C. Unbound complexes were washed away, and precipitated proteins were dissociated by heating (100°C) for 5 minutes in SDS sample buffer with mercaptoethanol. The protein sample was separated by SDS-PAGE in a 5% polyacrylamide gel and electroblotted to a PDVF membrane. The blots were incubated with specific primary mAbs against LMα5 chain and detected with the HRP-conjugated secondary Ab (Dako). A generic ECL reagent kit (GE Healthcare Biosciences) was used for film development.

### Immunohistochemistry

Specimens of normal human kidney and skin were obtained from surgical operations and retrieved from the files of the Institute of Biomedicine/Anatomy of the University of Helsinki with institutional permission and approval from the Ethics Committee of the Hospital District of Helsinki and Uusimaa (#319/E0/2006). The material was derived from nonessential parts of clinical specimens used for diagnostic purposes. No separate consent is needed as long as the material is handled as anonymous. The exempt from the informed consent is based on the permit/licence granted by the National Supervisory Authority for Welfare and Health of Finland (# 5280/04/046/06). For indirect immunohistochemistry, 6-μm frozen tissue sections were fixed in acetone at −20°C for 10 minutes and exposed first to mAbs and then to FITC-coupled goat anti-mouse IgG (Jackson Immunoresearch, West Grove, PA). After immunostaining, the specimens were embedded in sodium veronal: glycerol buffer (1∶1, pH 8.4) and examined with a Leica Aristoplan microscope equipped with appropriate filters (Leica, Bensheim, Germany).

### Cell adhesion and migration assays

In cell adhesion and migration assays KG1C glioma, BE melanoma and MDA-MB-231 carcinoma cells were used, as well as function-blocking integrin (INT) mAbs P1B5 (INT α3) (Millipore), GOH3 (INT α6) (R&D Systems, Abingdom, UK) and 13 (INT β1) (BD Biosciences), together with mAbs to LMα5 chain.

The cell adhesion assay was performed in 96-well flat-bottomed polystyrene plates (Maxisorp, Nunc) coated with either laminins 511 or 521 (20 µg/ml) for 1 hour at RT. Following three washes with sterile PBS and blocking with 1% BSA for 1 hour, 100 μl of cell suspension (10^6^ cells/ml in RPMI-1640 medium with 0.1% BSA) was added to the coated wells. Cell adhesion was allowed to progress for 60 min at 37°C. Then, the wells were washed 5 times with pre-warmed medium, and adhered cells were fixed with 4% formaldehyde for 30 min and stained overnight with 0,5% toluidin blue (Sigma-Aldrich). Finally, the plate was washed 5 times with deionized water and 100 μl of 2% SDS was added to each well to release the dye. A generic ELISA-reader (630 nm wavelength) was used to measure the optical density. To test the effect of mAbs, cells and coated wells were preincubated with integrin or laminin antibodies (20 μg/ml), respectively, for 30 min before interacting.

Cell migration was performed in polycarbonate Transwell culture inserts (Costar, Cambridge, MA, USA) with 8-µm pore size membranes in 24-well plates. The membrane, which separates upper and lower compartments, was coated on its bottom side with laminins 511 or 521 (20 μg/ml) for one hour at 37°C and then washed three times with sterile PBS. Thereafter, the inserts were blocked with 0,1% BSA for 30 minutes and meticulously washed 3–5 times with sterile PBS. Cells (1×10^6^ cells/ml) were pre-treated for 15–20 minutes with integrin-blocking mAbs (20 µg/ml) in RPMI 1640 and then 100 µl of this mixture was added to each insert. Alternatively, 20 µg/ml of laminin mAb was added to the cell suspension and to the lower chamber, which contained 600 µl RMPI-1640. Cells were allowed to migrate at 37°C for 18 hours and the membranes then were fixed in 2% glutaraldehyde and stained with haematoxylin. Following removal of cells from the upper side of the membrane with cotton sticks, cells attached to the lower side were counted using a microscope. Mean cell number of four different representative fields (400x) was calculated. Cell migration in the presence of mouse IgG was defined as 100% control.

### Binding of soluble α3β1 integrin to immobilized laminins 511 and 521 and effect of laminin mAbs

Direct receptor-ligand binding was measured by ELISA using recombinant human α3β1 integrin (R&D Systems) and recombinant human laminins 411, 511 and 521 (BioLamina). Briefly, 96-well Maxisorp plates (Costar) were coated overnight at 4°C with laminins at 5 µg/ml and, after blocking with 1% BSA, Mn^++^-stimulated integrin (at 2,5 μg/ml) was added for 1 hour. Following three washes, biotin-labeled goat anti-human integrin β1 antibody (R&D Systems) was added at 1 μg/ml for 1 hour more. After three additional washes, HRP-conjugated streptoavidin (Dako) was added at 1∶500 dilution and the enzyme activity was measured using orthophenylenediamine. The plate was analyzed using a generic ELISA reader (at 492 nm). Interfering mAbs at 20 μg/ml were added to the immobilized laminins before incubation with the integrin. Tris-buffered saline (TBS) was used throughout the assay, including in the washing buffer (TBS with 0.1% BSA and 0.02%) and in the integrin-stimulating buffer (washing buffer with 2 mM MnCl_2_).

### Statistical analysis

Results are presented as mean and standard deviation. Significant differences were determined using paired Student's *t*-test. For all analyses, p<0.05 was considered statistically significant: *, p<0.05; **, p<0.01; ***, p<0.001.

## Results

### Production and identification of novel mAbs to LMα5 chain

**Figure 1:**
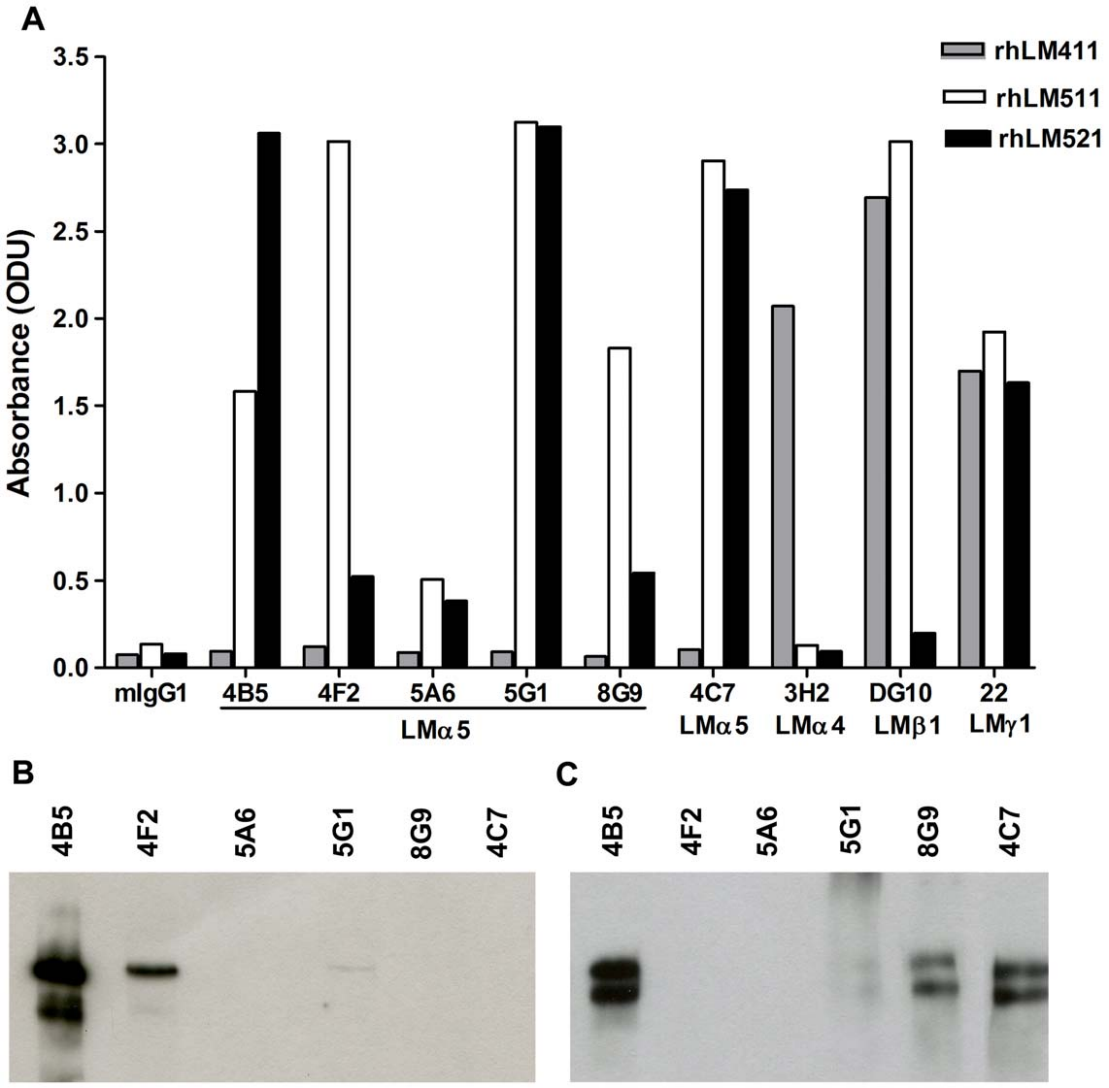
Reactivity of novel mAbs to LMα5 chain as measured by ELISA, Western blotting and immunoprecipitation. A) Reactivity of the antibodies with rhLMs 411, 511 and 521 by ELISA. B) Reactivity of the antibodies with rhLM511 by Western blotting under reducing conditions. C) Ability of the antibodies to immunoprecipitate laminin-511 from A549 cells' conditioned medium and detection of the LMα5 chain by Western blotting with mAb 4B5. Bands of 300/350 kDa corresponding to LMα5 chain were detected with some of the antibodies.

Considering the strong biological activity of laminin-511 and the poor availability of reagents against the LMα5 chain, new mAbs against this chain were generated using hybridoma technology. Laminin-511 isolated from the supernatant of A549 lung adenocarcinoma cells was used as the immunogen. Among the multiple laminin isoforms A549 cells selectively produce laminin-511 [14] (M. Patarroyo, unpublished data). The cells were found to express the LMα5 chain but not detectable amounts of other laminin α chains, and Western blot analysis of immunoprecipitates from the conditioned medium with mAbs to LMα5 chain under reducing conditions demonstrated presence of LMα5 (300/350 kDa), LMβ1 (230 kDa) and LMγ1 (220 kDa), but not LMβ2 (190 kDa), chains [14] (M. Patarroyo, unpublished data). The isolated preparation used for immunization consisted of 300–350 and 220–230 kDa polypeptides with more than 95% purity as demonstrated by silver staining of gels following electrophoresis under reducing conditions. After a preliminary selection, five clones were chosen for further study (4B5, 4F2, 5A6, 5G1, 8G9). By comparison of the reactivity against recombinant human laminin (rhLM) 511 and rhLM411 by ELISA, the novel mAbs were found to selectively react with the former but not with the latter (Fig. 1A), indicating specificity for the LMα5 chain, similarly to the prototype mAb 4C7. Accordingly, these mAbs also reacted with rhLM521. mAb 3H2 to LMα4 chain exclusively bound rhLM411, whereas mAb DG10 to the LMβ1 chain and mAb 22 to the LM**γ**1 chain reacted with rhLM411 and rhLM511 and with the three laminin isoforms, respectively, as expected. Similar level of reactivity of mAb 22 to the LMγ1 with immobilized rhLM411, rhLM511 and rhLM521 indicated a comparable degree of adsorption of the three laminin isoforms to the plastic surface (Fig. 1A).

### Ability of LMα5 chain-specific mAbs to react by Western blotting under reducing conditions and to immunoprecipitate

To further characterize the novel mAbs, their ability to recognize the LMα5 chain in Western blotting under reducing conditions was analyzed (Fig. 1B). Against rhLM511, mAb 4B5 strongly reacted with a polypeptide of 350 kDa. Less strong reactivity was obtained with mAb 4F2 and a faint labeling was detected with mAb 5G1. Other mAbs, including the prototype mAb 4C7, were non-reactive.

In additional studies, the ability of the novel mAbs to immunoprecipitate α5 laminins was tested in the culture supernatant of A549 cells. The immunoprecipitated material was visualized by Western blotting under reducing conditions by using mAb 4B5 (Fig. 1C). Similarly to the prototype mAb 4C7, mAbs 4B5 and 8G9 were highly effective in immunoprecipitation, followed by mAb 5G1. mAbs 4F2 and 5A6 were poor for immunoprecipitation.

### Immunohistological reactivity of newly developed mAbs to LMα5 chain with kidney and skin sections when compared to the prototype mAb 4C7

**Figure 2:**
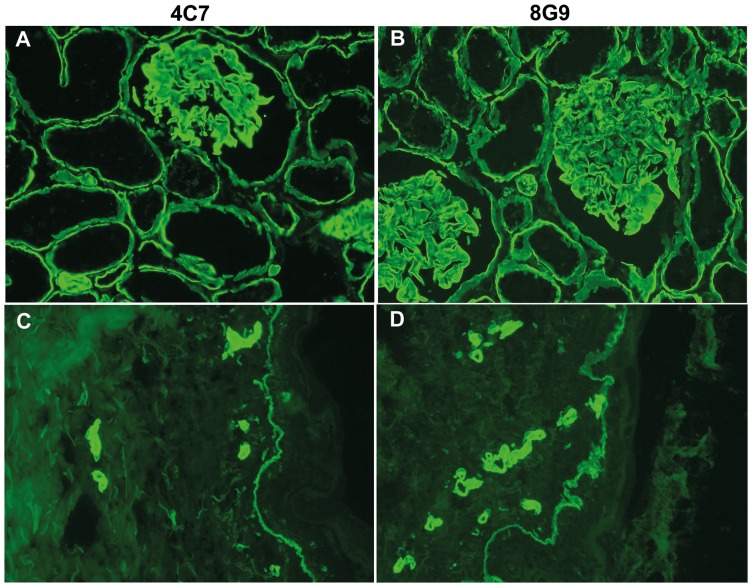
Immunohistochemical reactivity of mAb 8G9 in comparison to mAb 4C7 against LMα5 chain. (A) and (C), mAb 4C7; (B) and (D), mAb 8G9 against frozen sections of human kidney (A) and (B), and skin (C) and (D). The staining pattern of the two antibodies was indistinguishable. In kidney, the antibodies stained glomerular and tubular basement membranes, whereas in skin, reactivity with epidermal and vascular basement membranes was observed.

To confirm LMα5 chain specificity, reactivity of the novel mAbs with frozen sections of human tissues was analyzed by immunohistochemistry, and compared to that of mAb 4C7 (Fig. 2). The reactivity of all novel antibodies, but 4F2, was indistinguishable from that of mAb 4C7 as they readily stained glomerular and tubular BM in the kidney, and epidermal and vascular BM in the skin (shown for mAb 8G9 against kidney and skin in Fig. 2). Of interest, mAb 4F2 stained tubular, epidermal and vascular BMs but not, or only minimally, glomerular BMs indicating preferential reactivity with laminin-511 when compared to laminin-521 (data not shown). This pattern was originally observed by ELISA (Fig. 1A). The novel mAbs were non-reactive with formalin fixed paraffin embedded sections.

### mAb 8G9 to LMα5 chain strongly inhibits α3β1 and α6β1 integrin-mediated cell adhesion and migration on laminin-511 and, together with mAb 4C7, on laminin-521

**Figure 3:**
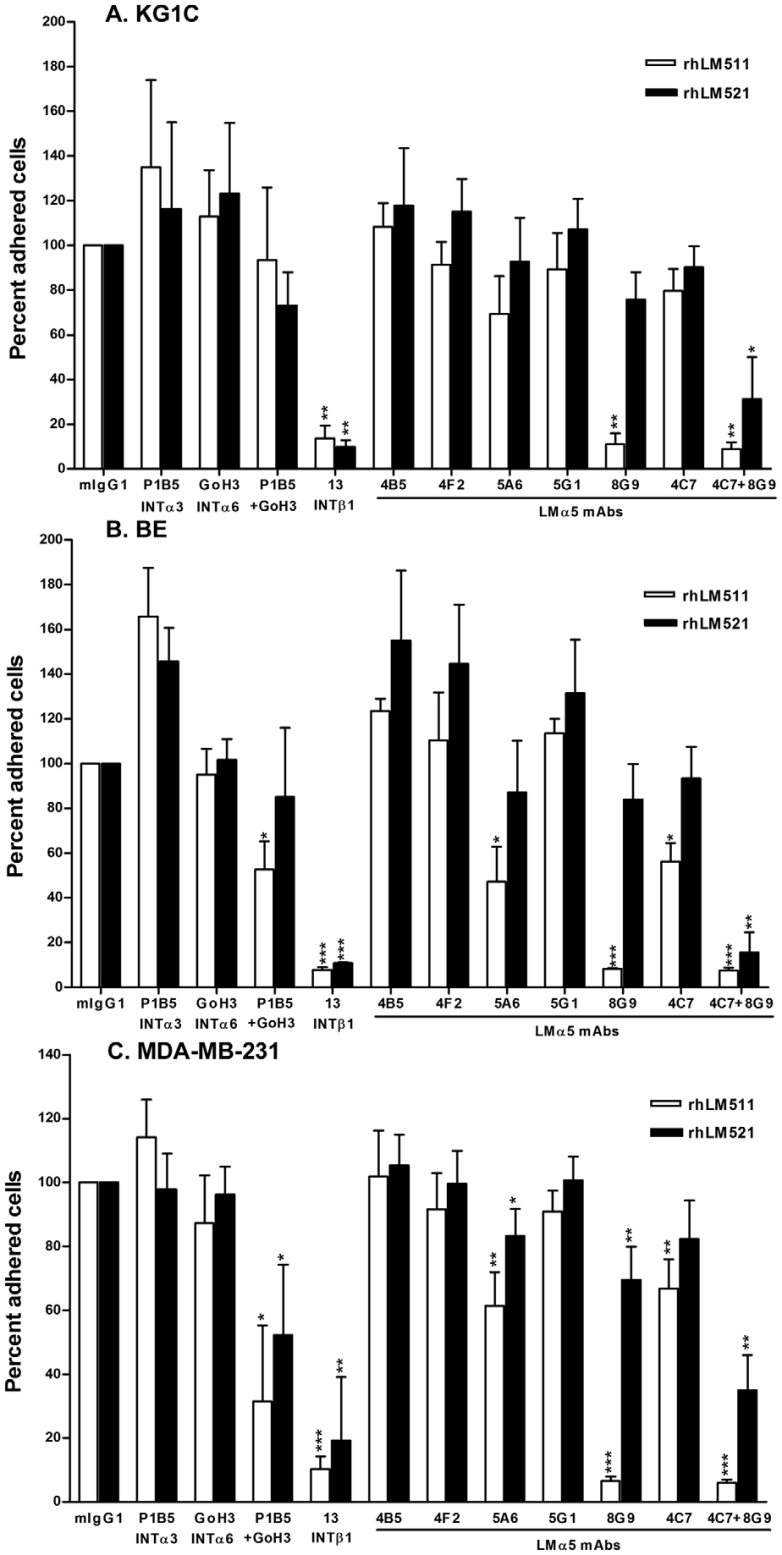
mAb 8G9 to LMα5 chain strongly inhibits α3β1/α6β1 integrin-mediated cell adhesion to laminin-511 and, together with LMα5 mAb 4C7, to laminin-521. A) KG1C glioma, B) BE melanoma and C) MDA-MB-231 breast carcinoma cells were used. Cells were allowed to adhere to surfaces coated with rhLMs 511 or 521 (20 μg/ml) for 1 hour at 37°C. Statistical analyses (Student's *t*-test) including mean and SD were calculated, as well as level of significance comparing antibodies to mouse IgG (*, p<0.05; **, p<0.01; ***, p<0.001).

In further studies, the function-blocking activity of the novel mAbs was analyzed in cell adhesion to rhLM511 and rhLM521 (Fig. 3). These laminins were strongly adhesive for KG1C glioma cells (OD values 1.48 and 1.39 for rhLM511 and rhLM521, respectively), BE melanoma cells (OD values 1.65 and 1.56 for rhLM511 and rhLM521, respectively) and MDA-MB-231 breast carcinoma cells (OD values 1.82 and 1.85 for rhLM511 and rhLM521, respectively), when compared to human serum albumin (HSA) (OD values 0.10, 0.10 and 0.09 for KG1C, BE and MDA-MB-231 cells, respectively) and the cell adhesion was largely mediated by β1 integrins, preferentially α3β1 and α6β1. Most notably, mAb 8G9 practically abolished the adhesion of the three cell lines on rhLM511, whereas mAb 5A6 and the prototype mAb 4C7 partially reduced the adhesion of melanoma and breast cancer cells. In contrast, mAb 8G9 was slightly inhibitory on rhLM521 when tested alone, but its blocking effect was strongly synergized with mAb 4C7 in the three cell lines.

**Figure 4:**
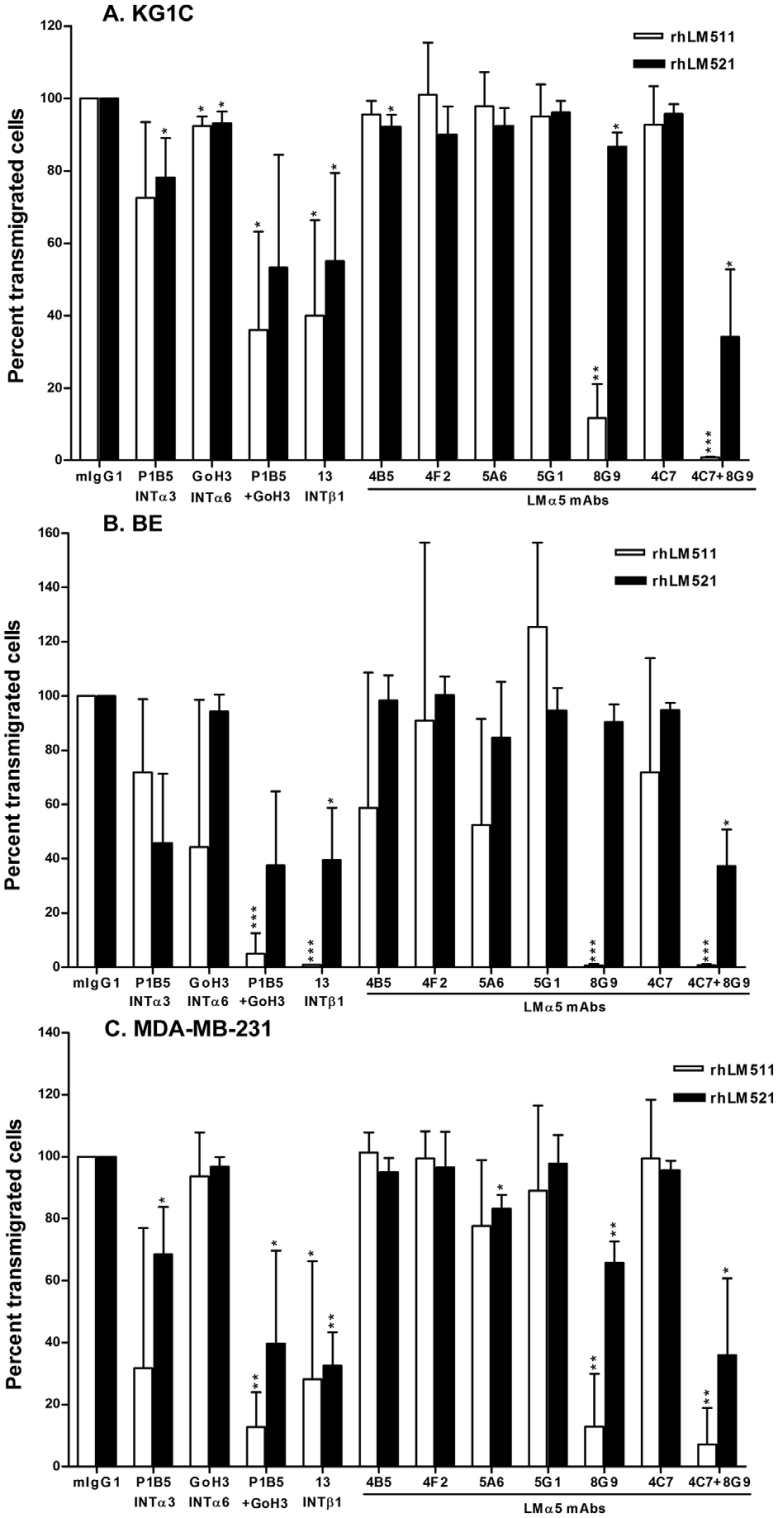
mAb 8G9 to LMα5 chain strongly inhibits α3β1/α6β1 integrin-mediated cell migration on laminin-511 and, together with LMα5 mAb 4C7, on laminin-521. A) KG1C glioma, B) BE melanoma and C) MDA-MB-231 breast carcinoma cells were used. Cells were allowed to migrate for 18 hours at 37°C through 8-μm pore-membranes coated with rhLMs 511 or 521 (20 μg/ml) on the lower surface. Statistical analyses (Student's *t*-test) including mean and SD were calculated, as well as level of significance comparing antibodies to mouse IgG (*, p<0.05; **, p<0.01; ***, p<0.001).

In cell migration assays, rhLM511 and rhLM521 strongly promoted migration of glioma cells (161 and 174 cells/field, respectively), melanoma cells (88 and 203 cells/field, respectively) and carcinoma cells (190 and 198 cells/field, respectively) when compared to HSA (1 cell/field for all cell lines). As for cell adhesion, cell migration was mediated via α3β1 and α6β1 integrins, and mAb 8G9 largely inhibited the migration of the three cell types on rhLM511 (Fig. 4). Also similarly to cell adhesion, mAb 8G9 alone slightly reduced cell migration on rhLM521, but largely inhibited cell migration on this laminin isoform when combined with mAb 4C7. mAb 8G9 did not inhibit β1-integrin mediated cell migration on other extracellular matrix proteins (data not shown).

### mAb 8G9 to LMα5 chain interferes with the binding of isolated α3β1 integrin to laminins 511 and 521

**Figure 5:**
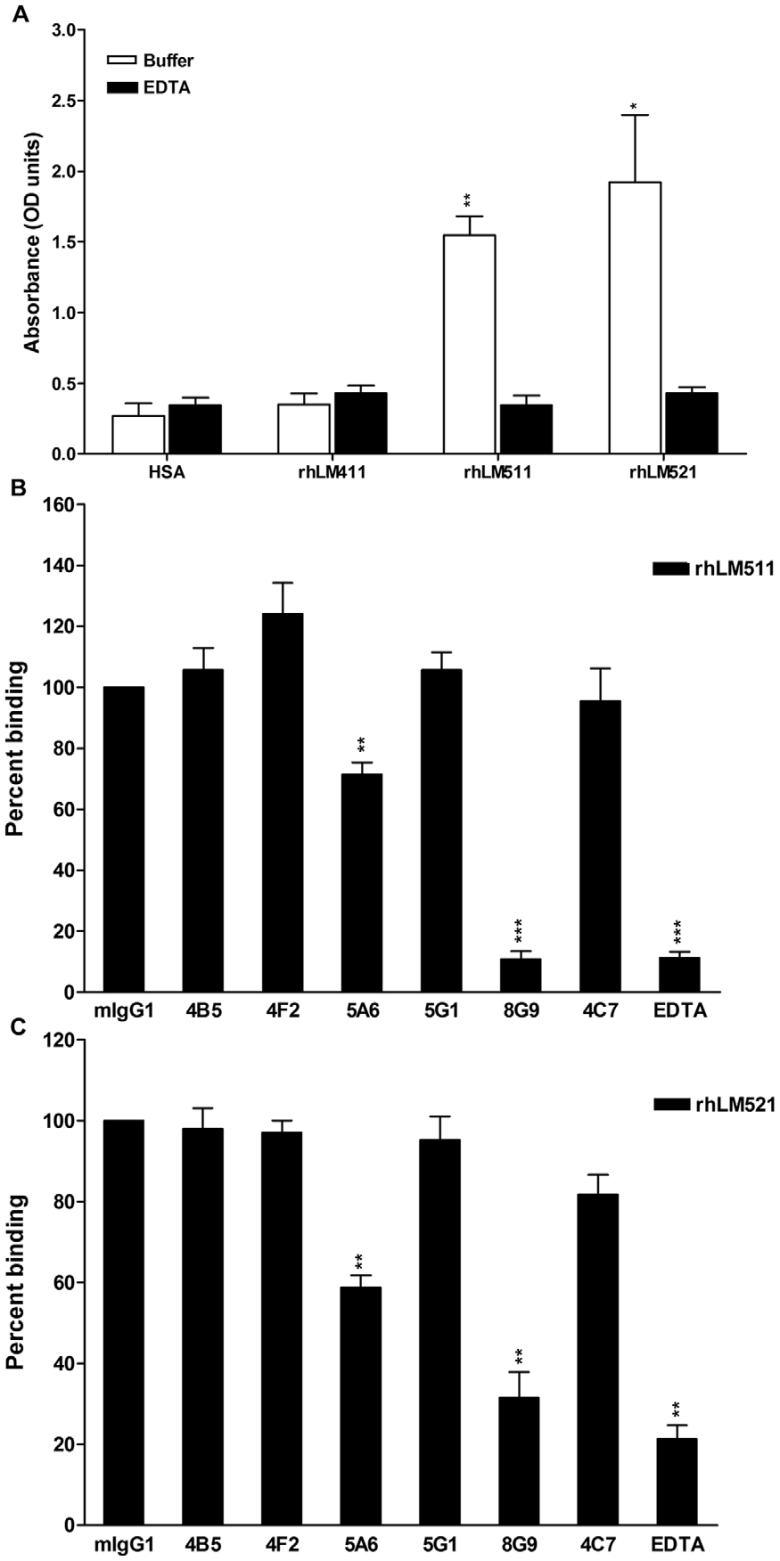
mAb 8G9 to LMα5 chain strongly inhibits binding of isolated α3β1 integrin to immobilized laminins 511 and 521. A) α3β1 integrin binds rhLMs 511 and 521, but not rhLM411. B) Effect of mAbs to LMα5 chain on the binding of α3β1 integrin to rhLM511. C) Effect of mAbs to LMα5 chain on the binding of α3β1 integrin to rhLM521. Statistical analyses (Student's *t*-test) including mean and SD were calculated, as well as level of significance of integrin binding to rhLMs 411, 511 and 521 when compared to HSA (A), and effect of antibodies when compared to mouse IgG on integrin binding to rhLMs 511 (B) and 521 (C) (*, p<0.05; **, p<0.01; ***, p<0.001).

To investigate whether mAb 8G9 and other LMα5 antibodies could hinder the physical interaction of integrins with laminins, the effect of the antibodies on the binding of isolated recombinant α3β1 integrin to immobilized rhLM511 and rhLM521 was measured by ELISA (Fig. 5). The integrin readily bound the two α5 laminins to a similar extent, but not rhLM411 or albumin, and the binding was totally inhibited by EDTA (Fig. 5A). Among the LMα5 antibodies, mAb 8G9 practically abolished the integrin interaction with rhLM511, whereas mAb 5A6 exerted a modest, but statistically significant, inhibitory effect (Fig. 5B). All other antibodies, including mAb 4C7, were practically inactive. Similar results were obtained with rhLM521 (Fig. 5C).

### mAb 8G9 defines an epitope located at or near to the globular domain of laminin-511

**Figure 6:**
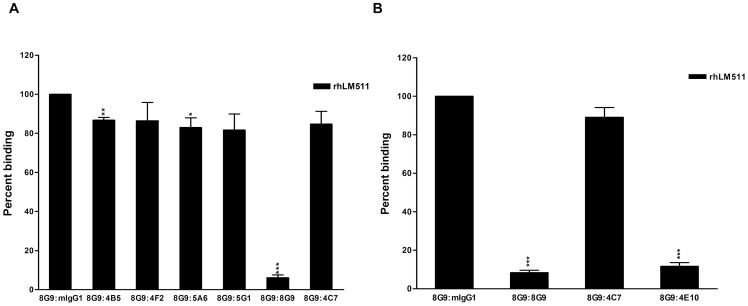
mAb 4E10 to a LMβ1 chain epitope near the globular domain of laminin-511 hinders the binding of mAb 8G9. A) mAb 4C7 and other LMα5 antibodies minimally affect the binding of mAb 8G9 to rhLM511. B) mAb 4E10 largely competes with the binding of mAb 8G9 to rhLM511. Statistical analyses (Student's *t*-test) including mean and SD were calculated, as well as level of significance of the effect of the mAbs on the binding of biotinylated-8G9 to rhLM511 when compared to mouse IgG. Competing antibodies were used at 50x higher concentration than biotinylated-8G9 (*, p<0.05; **, p<0.01; ***, p<0.001).

The blocking activity of mAb 8G9 in the cell adhesion and migration mediated by α3β1 and α6β1 integrins and in the binding of isolated α3β1 integrin to α5 laminins suggested localization of its target epitope at, or near, the globular domain of the LMα5 chain; both integrins are known to recognize this specific region [33]. To more closely determine the localization of the epitope, competition of binding of biotinylated-mAb 8G9 to immobilized rhLM511 with other mAbs to LMα5 chain was analyzed by ELISA (Fig. 6). Non-labeled mAb 8G9 was highly competitive, as expected, but all other antibodies, including mAb 4C7 which binds the LG1 module of the globular domain [41], were practically inactive (Fig. 6A). In contrast, mAb 4E10, against a LMβ1 epitope located close to the globular domain of laminin-511 [38], strongly interfered with the binding of biotinylated-mAb 8G9 (Fig. 6B).

## Discussion

In the present study, we report development of five novel mAbs to LMα5 chain. These antibodies were highly effective for immunohistochemistry, Western blotting and immunoprecipitation. We also describe, for the first time, a mAb (8G9) that efficiently inhibits integrin-dependent adhesion and migration of a variety of cancer cells on laminin-511 and, when combined with mAb 4C7, on laminin-521. The strong adhesion- and migration-promoting activities of the α5 laminins, described for the first time for laminin-521 in the present work, were found to be mediated by α3β1 and α6β1 integrins, in accordance with the known laminin isoform-specific binding of integrins [31]. Furthermore, we report that mAb 8G9 binds at, or near, the globular domain of the LMα5 chain and that the antibodies hinder the binding of α3β1 integrin to the α5 laminins.

The specificity of the mAbs was first established by ELISA based on their reactivity with rhLM511, but not rhLM411, which differ only in the α chain. Subsequently, the LMα5 specificity of the mAbs was confirmed by Western blotting (4B5, 4F2, 5G1) and immunoprecipitation (4B5, 5G1, 8G9). In contrast to the prototype mAb 4C7, mAb 4B5 strongly reacted by Western blotting and, similarly to 4C7, mAbs 4B5 and 8G9 were highly effective for immunoprecipitation. The mAb 15H5, produced against the recombinant domain IIIb of the short arm of the human LMα5 chain, has also been reported to work for Western blotting under reducing conditions [14]. Furthermore, all five antibodies readily labeled frozen tissue sections. Of interest, the immunohistochemical reactivity of mAb 4F2 differed to some extent from that of the others. This antibody reacted with epidermal, vascular and tubular BMs, as the other antibodies did, but in contrast did not stain the glomerular BM. Because the latter BM contains laminin-521 and the other BMs express laminin-511, mAb 4F2 could be laminin-511-specific and differentiate between the two α5 laminins. This inference was also supported by its selective reactivity with rhLM511 when compared to rhLM521 by ELISA. Theoretically, the LMβ2 chain, but not LMβ1, might hinder binding of the antibody to the long arm of the LMα5 chain. Lack of reactivity of the antibodies with formalin fixed paraffin embedded sections indicated destruction of the corresponding epitopes by the formalin fixation.

In contrast to laminin-511, the biological role of laminin-521 is poorly known. This laminin isoform, which is found in the glomerular BM in kidney, in the neuromuscular synaptic cleft in skeletal muscle, in blood platelets and in other tissues [20], [46] was, at least, as active as laminin-511 in promoting cell adhesion and migration via α3β1 and α6β1 integrins. In addition, it was bound to a slightly higher degree by α3β1 integrin than laminin-511. These results agree with a recent report from Taniguchi et al. [47] describing more avid binding of β2 laminins to integrins such as α3β1 when compared to β1 laminins.

Among our findings, the strong inhibitory effect of mAb 8G9 on cell adhesion and migration was remarkable. The mAb 4C7 has been previously reported to be a function-blocking mAb [34], [38], [40], [41], but our data demonstrated the superiority of mAb 8G9. Considering that the cell adhesion and migration on laminins 511 and 521 was mediated by α3β1 and α6β1 integrins and that these integrins recognize the globular domain of the LMα5 chain [33], it was plausible to assume that mAb 8G9 interacted with, or near, the globular domain and recognized a site at, or near, the binding sites of these two integrins. The former was proven by the ability of mAb 4E10, which binds to a LMβ1 epitope close to the globular domain [38], to compete out the binding of mAb 8G9. Moreover, interference of soluble integrin binding to the α5 laminins was demonstrated for α3β1. The latter studies with soluble α3β1 integrin also confirmed the isoform-specific interaction with laminins, as this integrin bound laminins 511 and 521, but not laminin-411 [31]. Whether mAb 8G9 and the other novel mAbs hinder the binding of α6β1, α6β4, α7β1 and other integrins to laminins 511 and 521 is currently under investigation.

The more effective inhibition of cell adhesion and migration on laminin-511, when compared to laminin521, by mAb 8G9 may be explained by its preferential binding to the former laminin isoform as detected by ELISA. This preferential behaviour may be due to modulation of the antibody binding by the C-terminal region of the laminin β1 and β2 chains, as demonstrated for integrin binding [47]. In contrast to mAb 8G9, which abolished the binding of soluble α3β1 integrin to α5 laminins, mAb 4C7 was practically inactive in this assay. However, the latter mAb is known to bind the LG1 module of the globular domain and to inhibit binding of α6β1 integrin to laminin-511 [41]. Together, these findings indicate that the binding site(s) of these two integrins, known to be located at LG1-3 modules of the LMα5 chain globular domain [33], are close to one another but clearly distinct. mAbs 8G9 and 4C7 appear to inhibit preferentially binding of α3β1 and α6β1 integrins, respectively, and the two antibodies exert a complementary inhibitory effect on cells that express both integrins.

The strong inhibitory effect of mAb 8G9 on adhesion and migration on laminin-511 and, together with mAb 4C7, on laminin-521 in cells derived from different tumor types, namely glioma, melanoma and breast carcinoma, was striking considering that their integrin repertoire was not identical [22], [24], [48]. However, all cell lines expressed and used α3β1 and α6β1 integrins [22], [24], [48], suggesting a rather common mechanism in tumor cell adhesion and migration and, probably, invasiveness.

Altogether, the present results indicate that the five novel mAbs recognize five different epitopes on the LMα5 chain, distinct from the one defined by mAb 4C7. These reagents may constitute valuable tools for a better understanding of the role of α5 laminins in cell and tumor biology. mAb 8G9 may be the LMα5 antibody homologue of function-blocking mAbs BM165 and CM6 against the globular domain of the human and rat LMα3 chains, respectively [49], [50]. Recently, mAbs to the globular domain of the human LMα3 chain were shown to inhibit growth of human epithelial tumors grafted in mice [51], [52]. Likewise, antagonists of α5 laminins may inhibit tumor invasion and metastasis and have therapeutic potential in malignant diseases. Since immune cells similarly interact with α5 laminins in adhesion and migration, these inhibitory agents may also have a beneficial effect in inflammatory disorders [23], [32].
